# Nanofiber formation as a promising technology for preservation and easy storage of extracellular vesicles

**DOI:** 10.1038/s41598-022-25916-6

**Published:** 2022-12-20

**Authors:** Krisztina Németh, Adrienn Kazsoki, Tamás Visnovitz, Balázs Pinke, László Mészáros, Edit I. Buzás, Romána Zelkó

**Affiliations:** 1 Department of Genetics Cell and Immunobiology, Semmelweis University, Nagyvárad Square 4, Budapest, 1089 Hungary; 2 ELKH-SE Translational Extracellular Vesicle Research Group, Budapest, Hungary; 3 University Pharmacy Department of Pharmacy Administration, Semmelweis University, Hőgyes Endre Street 7-9, Budapest, 1092 Hungary; 4 Department of Plant Physiology and Molecular Plant Biology, Eötvös Loránd University, Pázmány Péter Sétány 1/C, Budapest, 1117 Hungary; 5 Department of Polymer Engineering, Faculty of Mechanical Engineering, Budapest University of Technology and Economics, Műegyetem Rkp. 3, Budapest, 1111 Hungary; 6 ELKH-BME Research Group for Composite Science and Technology, Műegyetem Rkp. 3, Budapest, 1111 Hungary; 7 HCEMM-SU Extracellular Vesicle Research Group, Budapest, Hungary

**Keywords:** Nanoscience and technology, Nanomedicine, Nanoscale materials, Techniques and instrumentation

## Abstract

Extracellular vesicles (EVs) are cell-derived, membrane-enclosed particles with the potential for a wide range of future therapeutic applications. However, EVs have almost always been administered by direct injection, likely hindering their efficacy because of rapid clearance from the injection site. The present study aimed to incorporate medium-sized extracellular vesicles (mEVs) into fast-dissolving electrospun polyvinylpyrrolidone-based nanofibers to explore the storage-dependent structure–activity relationship of the resulting nanofibrous formulations. Aqueous polyvinylpyrrolidone-based precursor solutions were selected for the electrospinning process. The presence of EVs in the electrospun samples was confirmed by transmission electron microscopy, flow cytometry, and confocal laser scanning microscope. The results indicate that the fibrous structure of the samples was preserved until the end of the 12-week storage period. Furthermore, regardless of the storage temperature (4 °C or room temperature), nanofibers and nanofiber-associated EVs were present throughout the experimental period. Incorporating EVs into a stable solid polymeric delivery base could preserve their stability; meanwhile, according to the characteristics of the polymer, their targeted and controlled release can be achieved.

## Introduction

Extracellular vesicles (EVs) are small lipid bilayer-delimited vesicles that differ in size and biogenesis, are naturally released from multivesicular bodies/amphisomes or the plasma membrane, and then enter the bodily fluids^1^. In addition to their physiological role in cellular homeostasis, intercellular communication, and immune response as natural biocompatible carriers of bioactive substances, recent interest has turned towards their use as a means of drug delivery^2,3^. EVs can allow for the efficient internalization of vesicle-encapsulated drugs^4,5^. EVs can be taken up by recipient cells, which enables their application both as primary therapeutics or drug delivery vehicles.

Mesenchymal stem cell-derived EVs (MSC-EV) have shown promising potential in tissue regeneration. However, their therapeutic potential is limited by their rapid depletion and short half-life^6^. Various approaches for exogenous incorporation of drugs into isolated EVs have been described, ranging from simple incubation, lipophilic molecules, or active loading techniques of hydrophobized compounds, such as repeated freeze–thaw and permeabilization with saponin, extrusion, ultrasonication, and electroporation^7^. Molecules encapsulated in EVs can be functionally active biological substances, including proteins, mRNA, and miRNA, capable of transmitting signals to surrounding target cells as well as to distant organs via blood and lymphatic vessels^8^. The structural complexity of biologics can also pose extra formulation and delivery challenges, especially regarding stability. To reach the target tissue, EV can be administered via different routes, such as intravenous, intraperitoneal, oral, intranasal, and subcutaneous. However, EVs have almost always been administered as direct injections, which is likely to inhibit their effectiveness due to rapid efflux from the injection site^9^. To minimize the toxic action of EVs in non-target organs, and to maximize the intended therapeutic effects, the isolated EVs should be incorporated into biomaterials that can control their release.

EVs can be separated from the conditioned medium of cell cultures. Concerning the storage of conditioned medium, the International Society for Extracellular Vesicles recommends (MISEV2018) the storage of EVs in phosphate-buffered saline at -80 °C in siliconized vessels to prevent adherence of EVs to surfaces^10^ at − 80 °C.

Recently, PBS containing HEPES, human serum albumin and trehalose^11^. However, the use of this storage condition may be limited by cost and transport challenges.

Thus, alternative solutions are needed to improve the storage stability of EVs^12^. Based on some previous studies^13,14^ it can be concluded embedding EVs in nanofibers could be a good solution to overcome their stability problems. Thecells have been shown to remain viable after electrospinning, and the resulting cell-filled fibers can be used for a variety of therapeutic applications. Trindade et al.^15^ developed fast-dissolving scaffolds to allow a rapid evaluation of EV-loading. They found that the choice of solvent is especially relevant when processing biological material in single-fluid electrospinning. Trifluoroethanol was used instead of ethanol since it is a gentler solvent on biological samples^16^. The electrospinning is a fast, cost-effective, scalable, room-temperature technology can form complex nanofibrous structures and good choice for formulation of sensitive actives.

In our project, as the base of the electrospun nanofibrous formulation polyvinylpyrrolidone (PVP) was chosen. The PVP is an inert, non-toxic, and biocompatible polymer making it a versatile excipient for both conventional formulations and novel controlled or targeted delivery systems, serving as a binder, coating agent, suspending agent, pore-former, solubilizer, stabilizer, etc. PVP with different molecular weights and concentrations is used in a variety of formulations for different purposes. Due to the water-solubility, hydrophilicity, and hydrogen bond-forming ability can improve the bioavailability and stability of the active pharmaceutical ingredients, can improve the physicochemical stability of the preparations, can adjust the release rate of drugs, and can prolong the in vivo circulation time of liposomes^17,18^.

Nanofibrous samples containing mEVs can be dissolved ex tempore, thus the good water-soluble property of the matrix is essential. In addition, the PVP is a good choice for the fiber formation process as it has excellent electrospinnable property also^19–22^. Isotonic solution is needed to preserve the structure of the EVs, but the surface tension of the solution increases, making the stable fiber formation process more difficult. There are other hydrophilic, biocompatible polymers, e.g., cellulose derivatives, which are also commonly used in pharmaceutical formulations, but fiber formation from their aqueous solutions is difficult, let alone from their isotonic solutions^23–25^. However, cellulose nanofibers naturally form nanoporous networks; therefore, it can potentially entrap nanosize EVs close to the cellulose nanofiber pore's size. Moreover, different chemistry interactions such as via hydrogen bonding, electrostatic forces, or other specific interactions might enhance the isolation efficiency^26^.

The increased understanding of how to best store the isolated and processed therapeutic EVs would allow EV-based approaches to meet their full versatile potential as a new class of promising therapeutics and drug carriers. Therefore, the present work aims to investigate the incorporation of EVs into a biodegradable fast-dissolving electrospun PVP-based nanofibers prepared from an aqueous precursor solution and to explore the storage-dependent structure–activity relationship of the resulting nanofibrous formulations. Our characterization of the nanofibrous formulations included the evaluation of morphology, physicochemical properties, and storage stability.

## Materials and methods

### Materials

As the base of the formulation the biodegradable, biocompatible well electrospinnable polymer polyvinylpyrrolidone (PVP, Kollidon K90, average molecular weight, M_w_ ~ 1,500,000 g mol^−1^ (Merck Ltd, Budapest, Hungary was chosen. Polysorbate 80 (Molar Chemicals Ltd., Budapest, Hungary) was used to decrease the surface tension of the solution and enhance the fiber formation ability of the isotonic precursor solution. The sodium chloride (Molar Chemicals, Budapest, Hungary) was used for the isotonization of the PVP-based precursor solution used for the fiber formation process. For the solution preparation distilled water of pharmacopeial grade without further purification was used.

### Separation of medium-sized extracellular vesicles (mEVs) from the conditioned medium of HEK293T-palmGFP cells

The HEK293T-palmGFP cell line was kindly provided by Charles Lai and Xandra Breakefield. The HEK293T-palmGFP cells were cultured in DMEM containing 10% (v/v) fetal bovine serum (FBS), 1000 U/L penicillin, 1000 U/L streptomycin, 2 mM L-glutamine, and 1 g/L glucose. Cells were maintained at 37 °C in a humidified atmosphere at 5% (v/v) CO_2_. The Mycoplasma infection status of the cell culture was monitored. The conditioned medium was collected 24 h after FBS deprivation from eight, 182.5 cm^2^ treated surface flasks (VWR, USA). The passage number ranged from 16 to 22, and the cell culture reached 80–90% confluence, representing 179 × 10^6^ ± 69.3 × 10^6^ cells per separation. During EV separation, cell viability was determined by trypan blue staining and flow cytometry, staining with TO-PRO-3 and Annexin V. During EV separation, the conditioned medium was centrifuged at 300 g at 4 °C for 10 min, and was filtered with a 5 μm cell strainer (Millipore, USA). The large-sized Evs were removed at 2000 g at 4 °C for 30 min. The mEVs were separated from the supernatant at 12,500 g at 4 °C for 40 min. The mEV pellet was washed once with NaCl-HEPES (10 mM, 12,500 g, 4 °C, 40 min). The mEV pellet was resuspended in 100 µL NaCl-HEPES (10 mM). 50 µL (1.6 × 10^9^ ± 6.7 × 10^8^ particles) was used to form mEV-containing PVP nanofibers (PVP-mEV), 42 µL (1.3 × 10^9^ ± 5.6 × 10^8^ particles) was used to generate free mEV control samples, and 8 µL was used to characterize the mEV fraction. The presence of mEVs was confirmed by transmission electron microscopy (TEM), flow cytometry, nanoparticle tracking analysis (NTA), protein and lipid quantification. The detailed parameters of the centrifugation steps are summarized in Table S1.

### Characterization of the mEV fraction based on the MISEV2018 guidelines

#### Examination of mEVs by transmission electron microscopy (TEM)

The morphology of Evs was examined by transmission electron microscopy (TEM). The samples were prepared with minor modifications based on the work of Théry and colleagues^27^. Briefly, 2–3 µL of the sample was placed on a formvar coated grid (Sigma, USA) and incubated for 10 min at room temperature (RT). The residual solution was removed and fixed with 2% glutaraldehyde for 10 min. The grids were washed three times for 5 min and then the contrast was increased with uranyl oxalate. The samples were further contrasted and embedded in a mixture of 4% (w/v) uranyl acetate and 2% (w/v) methylcellulose. Samples were tested with JEOL 1011 TEM (JEOL Ltd., Tokyo, Japan).

#### Analysis of mEVs by nanoparticle tracking analysis (NTA)

The size distribution, median size, and particle concentration of mEVs were determined using NTA. The measurement was performed with a ZetaView PMX-120 (Particle Metrix GmbH, Meerbusch, Germany) using ZetaVIEW software. The parameters of the measurement are summarized in Supplementary Table 2. The NTA was not suitable for the characterization of mEVs embedded into PVP fibers, because the number of detected particles increased significantly after the dissolution of neat PVP fibers (Fig. S1.).

### Determination of protein and lipid content of mEVs

Protein concentration was determined using a Micro BCA assay (ThermoFisher, USA) for which EV samples were prepared by 3–4 freeze–thaw cycles and sonication (10 min, 4 °C). Lipid content was measured by the sulfophosphovanillin lipid assay optimized by Visnovitz et al., 2019^28^.

### Characterization of EV marker expression by flow cytometry

The mEVs were characterized by CytoFLEX S flow cytometer (Beckman Coulter, USA) based on the detection of GFP, AnnexinV, CD81, and CD63. mEVs were diluted in Annexin Binding Buffer (AxBB, 10 mM HEPES, 0.14 M NaCl, 2.5 mM CaCl_2_) containing fluorochrome-conjugated AnnexinV or antibodies. The following reagents were used: AnnexinV-AF647 (1:200, Sony Biotechnology, USA), monoclonal anti-human CD81-PerCP-Cy5.5 (1:200, isotype: mouse IgG1, clone: 5A6, Sony Biotechnology, USA), monoclonal anti-human CD63-PerCP-Cy5.5 (1:200, isotype: mouse IgG1, clone: H5C6, Sony Biotechnology, USA), monoclonal mouse IgG1-PerCP-Cy5.5 (1:200, clone: MOPC21, Sony Biotechnology, USA). After 15 min of incubation, the samples were analyzed for one minute at a medium flow rate (30 µL/minute). The presence of mEVs was confirmed by Triton X-100 (0.1%, Molar Chemicals Kft., Budapest, Hungary) lysis. Raw data were processed with FlowJo-V10 software. All antibodies used in this experiment are listed in Table S3.

### Fiber formation and morphological investigation of the electrospun samples

Neat and mEV-loaded 15% (w/w) aqueous PVP polymer precursor solutions were made isotonic with NaCl and were used for the fiber formation process. To improve the fiber formation ability of the increased conductivity and surface tension of the isotonic solutions, polysorbate 80 was added in 1% (w/w).

A laboratory-sized electrospinning device (SpinSplit Ltd., Budapest, Hungary) was used to prepare the fibrous samples. Homogeneous precursor solutions placed in a plastic syringe (Luer lock syringe, Merck Ltd., Budapest, Hungary) were connected to a conventional emitter (22 G) with a Teflon tube. The syringe pump provided the continuous flow rate of the precursor solution. The applied voltage was 22–23 kV, the emitter–collector distance was 20 cm and the flow rate was 0.08 μL/sec during the fiber formation process. The electrospinning experiments were performed in a well-tempered room with a temperature of 22 ± 1 °C and 40 ± 5% humidity.

The morphological analysis of the electrospun samples was performed with a JEOL JSM-6380LA type scanning electron microscope (SEM) (JEOL Ltd., Tokyo, Japan), after sputtering with gold. Measurements were performed at an accelerating voltage of 15 kV and a sample distance of 10 mm. Samples formed on the aluminum foil were attached to copper ingots with double-sided carbon adhesive and then examined after gilding at multiple magnifications. The fiber diameters were measured with ImageJ software (US National Institutes of Health, Bethesda, MD, USA) and the average fiber diameter was calculated based on 100 different randomly selected individual fibers at 3500 × magnification.

### Investigation of the stability of free mEVs and mEVs embedded into PVP fibers (mPVP-mEVs) by flow cytometry

The stability of free mEVs and PVP-mEVs kept at 4 °C or RT was monitored twice a week for 12 weeks with a flow cytometer. The PVP-mEV nanotubes were removed from the surface of the aluminum foil using a spatula and the mass in an Eppendorf tube was measured using an analytical balance. PVP-mEV nanotubes were dissolved in AxBB (33.9 ± 3.7 µL AxBB/ 1 mg PVP-mEV). Blank PVP nanofibers were used as controls. The PVP, PVP-mEV and free mEV samples were diluted tenfold in antibody or AnnexinV-containing AxBB solution. Annexin V-AF647 (1:200, Sony Biotechnology, USA), monoclonal anti-human CD81-PerCP-Cy5.5 (1:200, isotype: mouse IgG1, clone: 5A6, Sony Biotechnology, USA), monoclonal mouse IgG1-PerCP-Cy5.5 (1:200, clone: MOPC21, Sony Biotechnology, USA) were used. After 15 min of incubation, the samples were diluted fivefold and measured with a CytoFLEX S flow cytometer (Beckman Coulter, USA) for one minute at a medium flow rate (30 µL/minute). Counting beads were used at 20-fold dilution (Count Check Beads-High, 2.436 × 10^5^ beads/mL, Sysmex, Germany) to determine the mEV number based on the following equation:

$$absolute\,mEV\,number = \frac{measured\,mEV\, number}{{measured\,bead\,number}} \times \frac{original\,bead\,number}{{sample\,volume}} \times dilution\,factor$$

The integrity of the mEVs was checked by lysis with TritonX-100 (0.1%). FlowJo-V10 software was used to analyze the raw data. All antibodies used in this experiment are listed in Table S3.

### Investigation of the stability of mEV-loaded PVP fibers by confocal laser scanning microscopy

The stability of PVP-mEV samples kept at 4 °C or RT was monitored four times a week for 12 weeks using a confocal laser scanning microscope. The PVP-mEV nanotubes were available on 24 × 32 mm coverslips (VWR, USA). The coverslips were examined with a Leica TCS SP8 confocal laser scanning microscope (Leica, Germany). The inverse arrangement of the microscope allowed us to detect the presence of mEVs associated with nanotubes at high resolution (immersion oil was used at the bottom and top). Samples were tested under the same settings: 63 × objective, 0.9% laser (488 nm) power, refractive index: 1.516, 16-line averaging, 3064 × 3064 resolution. The GFP fluorescence signal was detected by a hybrid detector with visible light at 300 V PMT.

### Statistical analysis

Values are shown as mean ± standard error. The statistical analysis and figures were prepared using GraphPad Prism 7.00 (USA) software. Two-way ANOVA and Tukey’s posthoc tests were used to compare the data. A *p*-value of less than 0.05 was accepted as statistically significant.

## Results and discussion

### Characterization of HEK293T-palmGFP-derived mEVs

**Figure 1:**
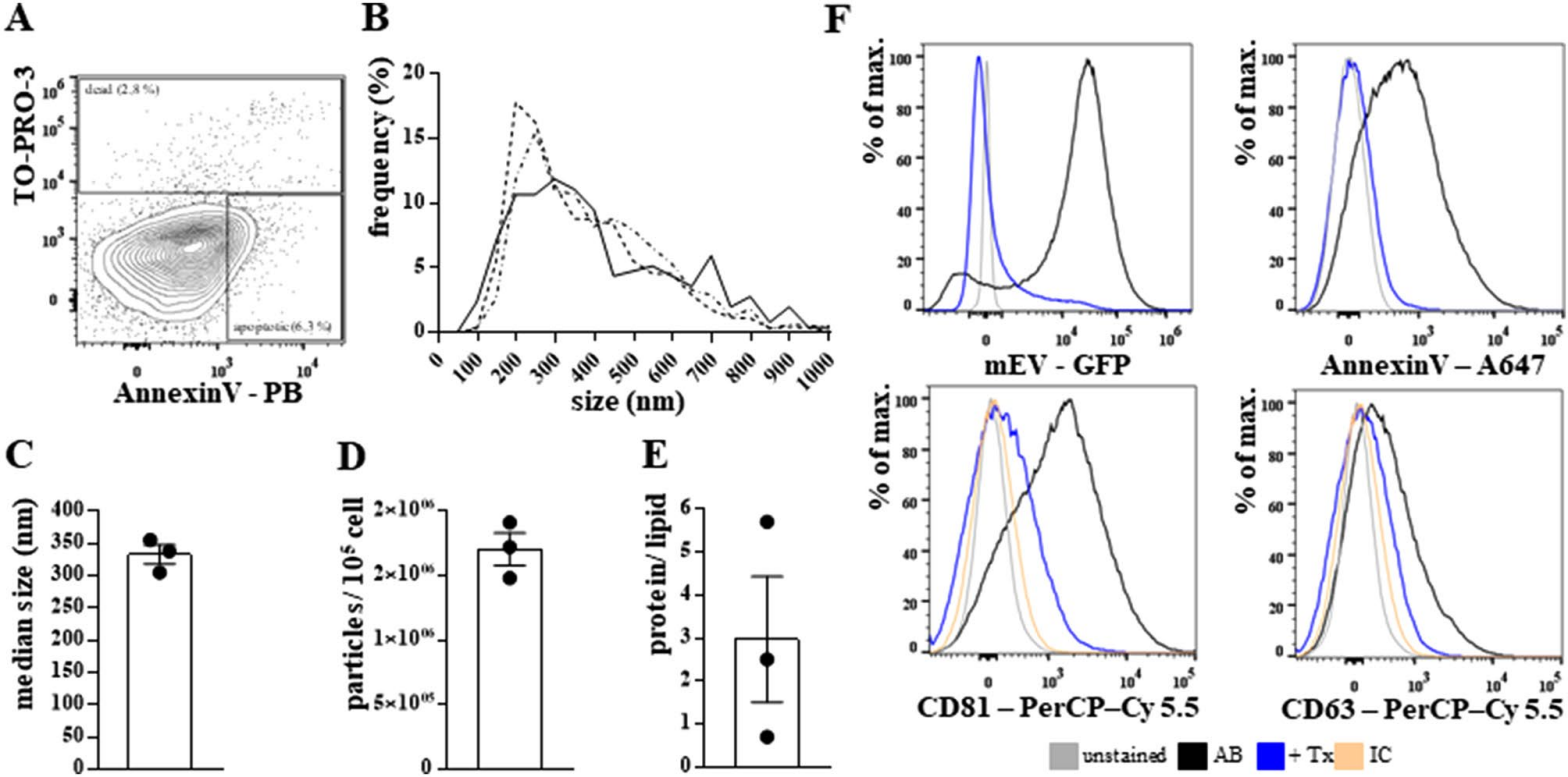
Characterization of HEK293T-palmGFP-derived free mEVs. The viability of the cells was analyzed by flow cytometry (**A**). We determined the size distribution (**B**), median size (**C**), and particle concentration (**D**) of mEVs by nanoparticle tracking analysis (NTA). We quantified the vesicles based on their protein and lipid content (**E**). The presence of GFP, externalized phosphatidyl-serine (stained with AnnexinV), CD81 and CD63 were detected by flow cytometry (**F**). (AB: antigen-specific staining with antibody, Tx: Triton X-100, IC: isotype control) (Figure was prepared using GraphPad Prism 7.00 for Windows, GraphPad Software, San Diego, California USA, http://www.graphpad.com).

The mEVs were separated from the conditioned medium of the HEK293T-palmGFP cells. Cell viability was 93.3 ± 0.9% (with trypan blue staining). Based on flow cytometric measurements, the proportion of the apoptotic (AnnexinV^+^) and secondary necrotic cells (AnnexinV^+^, TO-PRO-3^+^) was 3.9 ± 2.0% and 5.8 ± 2.1%, respectively (Fig. 1A). The size distribution of the mEVs was determined by NTA (Fig. 1B), based on which their median size was 332.4 ± 25.7 nm (Fig. 1C). The particle number was 1.7 × 10^6^ ± 2.2 × 10^5^/10^5^ cells (Fig. 1D). The protein/lipid ratio of mEVs was 3.0 ± 2.5 (Fig. 1E). Based on flow cytometric measurements, mEVs were GFP, AnnexinV, CD81, and CD63 positive and sensitive to Triton X-100 lysis (Fig. 1F).

### Morphological investigation of the mEV-loaded PVP fibers by electrospinning

**Figure 2:**
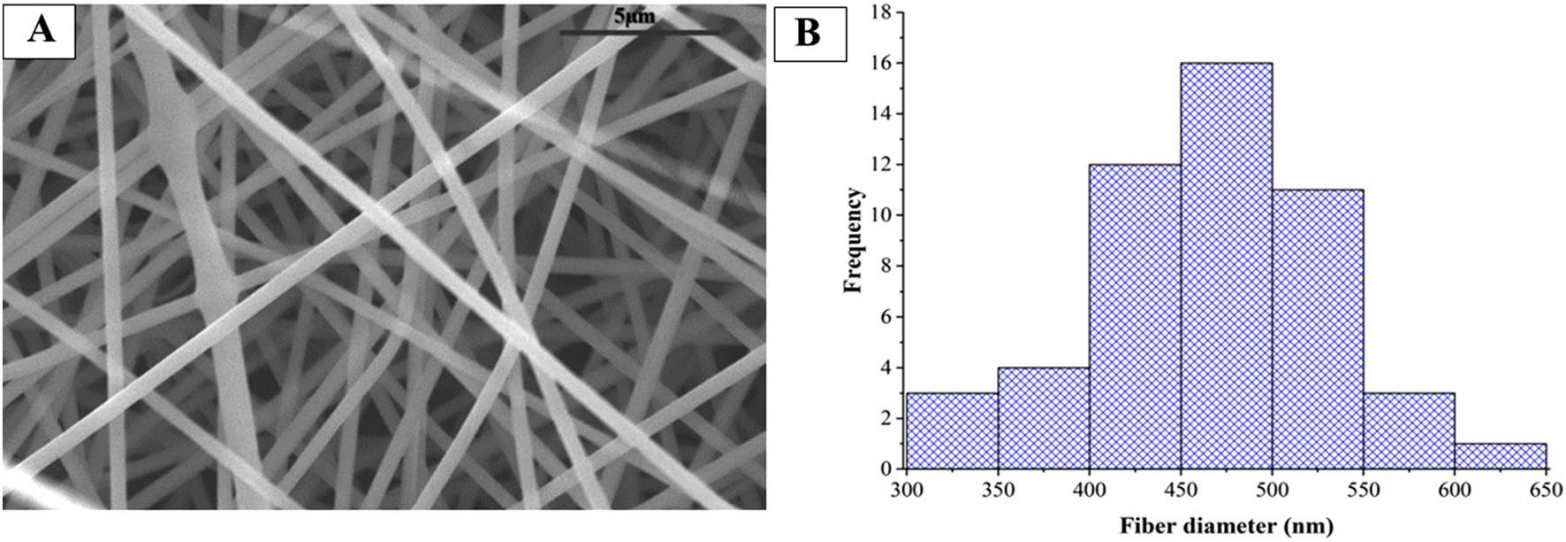
SEM image of the electrospun sample prepared from PVP-based precursor solutions containing medium-sized Extracellular Vesicles (mEV) (magnification: 2500×) (**A**) and fiber diameter distribution of sample (**B**).

The morphological characterization of the electrospun samples was performed by SEM. The image shows that the addition of vesicles to the precursor solution has no negative effect on the fiber formation (Fig. 2A) a clearly fibrous structure of 465 nm ± 62 nm average fiber diameter ± standard deviation with seemingly normal distribution was obtained (Fig. 2B). The surface of the fibers is smooth, indicating that both solution and fiber production parameters were appropriate for fiber production.

### Characterization of free mEVs and mEVs embedded into PVP fibers (PVP-mEV)

**Figure 3:**
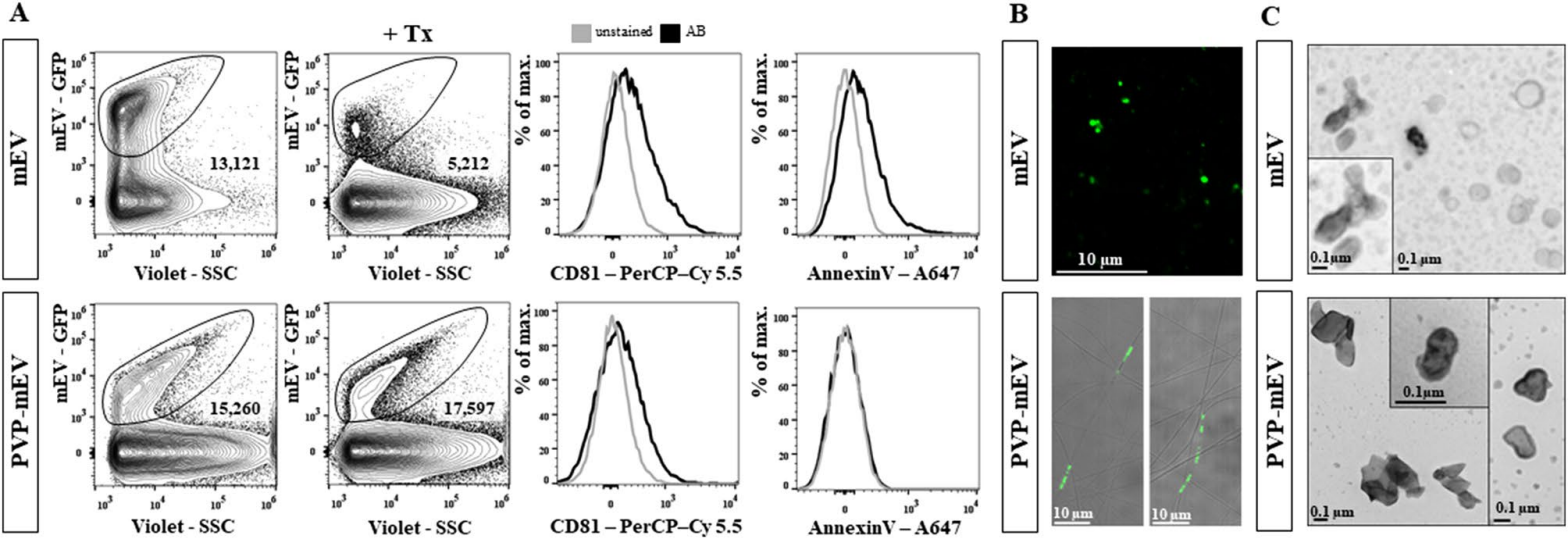
Comparison of free mEVs and mEV-loaded PVP fibers (PVP-mEVs). Free mEVs and PVP-mEVs were compared by flow cytometry based on GFP signal, TritonX-100 lysis, CD81 and phosphatidylserine (AnnexinV) markers (**A**). The mEVs were detected based on GFP fluorescence, while nanofibers were detected with visible light using a confocal laser scanning microscope (**B**). The presence and morphology of mEVs were confirmed by transmission electron microscopy (TEM) (**C**). (AB: antigen specific staining with antibody, Tx: Triton X-100).

Free mEV and PVP-mEV samples were characterized by flow cytometry, confocal laser scanning microscopy, and TEM (Fig. 3A–C). Compared to free mEVs, the GFP median fluorescence intensity (MFI) of PVP-mEVs decreased, and the standard deviation of the size distribution based on the Violet-SSC value increased. While free mEVs were sensitive to Triton X-100 lysis, the number of PVP-mEVs did not change significantly. Regarding the CD81 EV marker, free mEV showed a higher MFI value compared to PVP-mEVs. Free mEVs showed AnnexinV positivity. In contrast, PVP-mEVs were AnnexinV negative (Fig. 3A).

The presence of mEVs was detected by confocal laser scanning microscopy. In both samples, their presence was successfully detected based on the fluorescence signal of GFP. In case of PVP-mEV samples, it can be seen that mEVs are present in a nanofiber-associated form (Fig. 3B). The morphology of free mEVs and PVP-mEVs after the dissolution was characterized by TEM. We could identify mEVs in both samples (Fig. 3C).

The observed difference between the free mEV and the PVP-mEV samples can be explained by the fact that PVP can attach to the surface of lipid-based nanoparticles through hydrogen bonds^29^. It forms a macromolecular protective shell that provides stability for nanoparticles and can protect against aggregation^30^. PVP can reduce the binding of proteins to liposomes because of its electrical neutrality^31^. Since liposomes and EVs behave similarly in many ways, we assume that a PVP shell can be formed around mEVs. Due to the presence of the PVP shell, it may decrease the accessibility of CD81 and phosphatidylserine to antibodies and AnnexinV. As PVP increasesthe stability of mEVs, they have lower sensitivity to TritonX-100 lysis.

### Investigation of the stability of free mEVs and mEV-loaded PVP fibers (PVP-mEVs)

**Figure 4:**
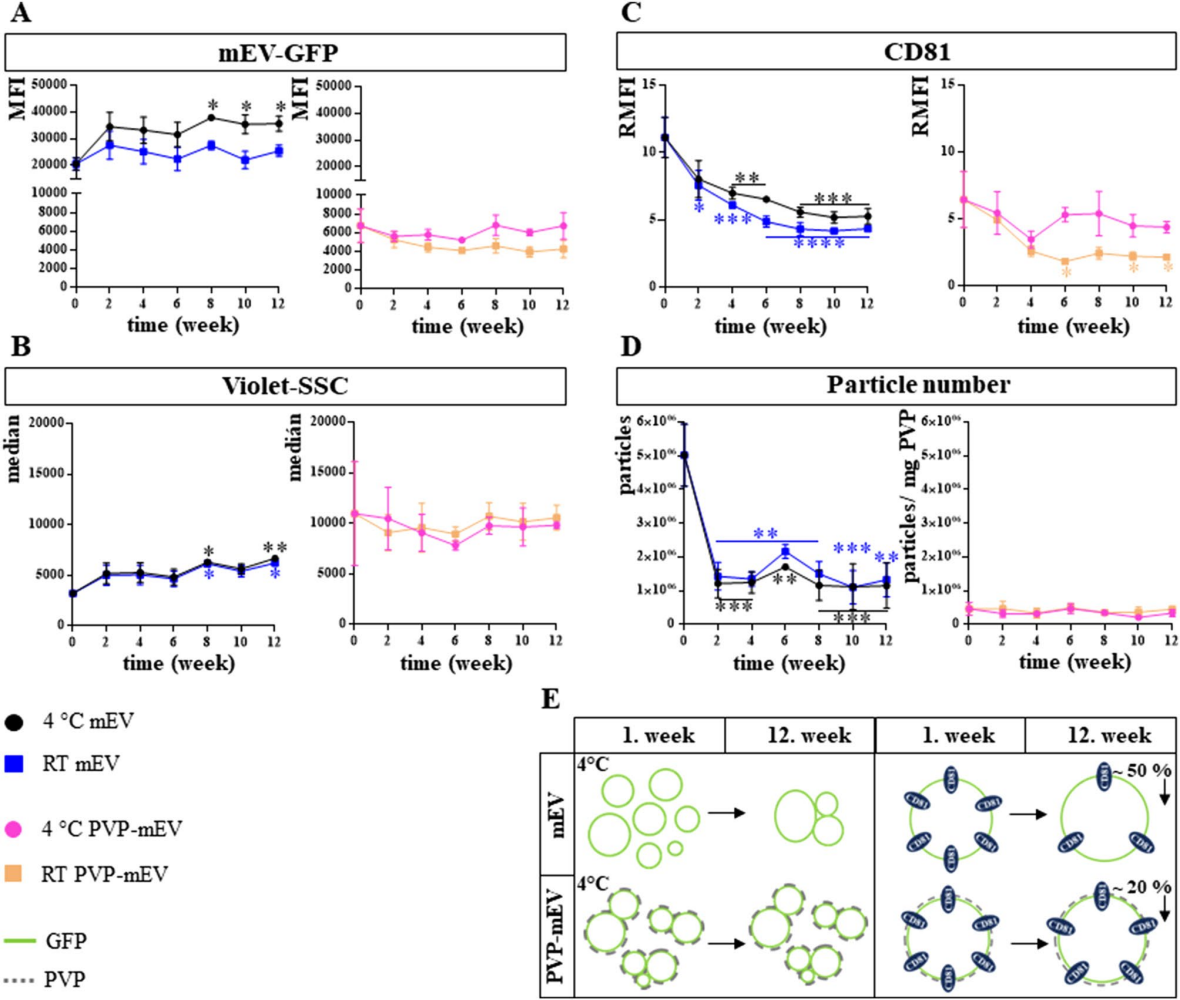
Stability investigation of free mEV and mEV-loaded PVP fibers (PVP-mEVs) by flow cytometry. The stability of free mEVs and PVP-mEVs kept at 4 °C or RT was monitored for 12 weeks, every two weeks by flow cytometry. The stability was investigated based on the membrane-bound GFP signal of mEVs (**A**), the Violet-SSC parameter correlating with the vesicle size (**B**), CD81 expression (**C**) and particle number (**D**). Schematic illustrations of the hypothesized mechanism of the changes observed for free mEV and PVP-mEV samples kept at 4 °C are summarized in panel (**E)**. n_mEV-GFP_ = 3, n_Violet-SSC_ = 3, n_CD81_ = 3, n_particle number_ = 2 (Figure was prepared using GraphPad Prism 7.00 for Windows, GraphPad Software, San Diego, California USA, http://www.graphpad.com).

The stability of PVP-mEVs and free mEVs maintained at 4 °C or RT was monitored for 12 weeks. Samples were analyzed every second weeks by flow cytometer based on GFP, Violet-SSC, CD81 parameters, and particle number. The GFP MFI and Violet-SSC median values of free mEVs were increased (Fig. 4A,B). In parallel, the relative MFI of CD81 decreased significantly as early as week 2. This trend was more pronounced for samples kept at RT (Fig. 4C). Irrespective of temperature, the calculated number of particles decreased after 2 weeks and remained low throughout the experiment (Fig. 4D). The mEVs bound to PVP nanofibers remained stable for 12 weeks based on the median value of Violet-SSC, GFP MFI, and particle number (Fig. 4A,B,D). The CD81 relative MFI of the samples kept at RT decreased significantly after 6 weeks, while the samples at 4° C remained stable (Fig. 4C). The relative MFI value of the CD81 isotype controls remained low during the duration of the experiment (Fig. S2). In addition to the median values, we also determined the robust coefficient of variation (rCV) value of the distribution curves (Fig. S3). Regarding the free mEVs, we saw a slight increase in all three parameters. In the case of PVP-mEVs, we saw an increasing trend for the CD81 marker in RT samples. Based on the similar H-bonding interaction described within PVP and the surface of the liposome, PVP fibers can form a macromolecular protective shell that provides stability for nanoparticles and can protect against aggregation (Fig. 4E). In addition, PVP can reduce the binding of proteins to liposomes because of its electrical neutrality. Since liposomes and EVs behave similarly in many ways, we assume that a PVP shell can be formed around medium-sized EVs. Due to the presence of the PVP shell, it may decrease the accessibility of CD81 and phosphatidylserine to antibodies and AnnexinV. As PVP increases the stability of medium-sized EVs, they have lower sensitivity to TritonX-100 lysis.

**Figure 5:**
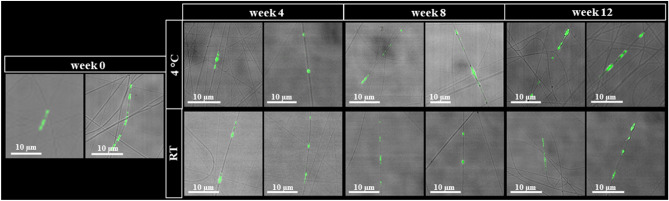
Investigation of the stability of mEV-loaded PVP fibers (PVP-mEV) by confocal laser scanning microscope. The stability of PVP-mEVs kept at 4 °C or RT was monitored for 12 weeks, every four weeks by confocal laser scanning microscope.

The stability of PVP-mEV fibrous samples kept at 4 °C or RT was monitored every four weeks for 12 weeks using a confocal laser scanning microscope (weeks 0, 4, 8, and 12). PVP nanofibers were detected with visible light, while EVs were detected by their GFP fluorescence. It can be clearly seen in Fig. 5 that the fibrous structure of the sample remained visible until the end of the 12-week storage period, furthermore, regardless of the temperature (4 °C or RT), nanofibers and nanofiber-associated GFP-positive mEVs were present throughout the experiment.

## Conclusion

The most promising store condition for EVs is − 80 °C; however, this condition may be limited by cost and transport challenges. An alternative EV preservation technique is lyophilization, but it is costly, and its reproducibility raises questions. Electrospinning offers a good alternative solution to improve the storage stability of vesicles. This fast, cost-effective, scalable, room-temperature technology can form complex nanofibrous structures and create a technology platform, making it a viable tool for formulating sensitive drugs. Embedding EVs in nanofibers enables solid-phase, stability-preserving storage of vesicles, opening up new horizons in research for therapeutic applications.

## Supplementary Information

Supplementary Information.

## Data Availability

All data generated or analysed during this study are included in this published article [and its supplementary information files].
