# Supplementary material for: Nanofiber formation as a promising technology for preservation and easy storage of extracellular vesicles
Source: Sci Rep. 2022 Dec 20;12:22012. doi: 10.1038/s41598-022-25916-6 (PMC9768167; doi:10.1038/s41598-022-25916-6)
Supplement: Supplementary file 1 — Supplementary Information. [file 41598_2022_25916_MOESM1_ESM.docx]

**Supplementary informations**

**Table S1. Separation of HEK293T-palmGFP-derived mEVs: detailed parameters of the centrifugation steps**

|  | **centrifuge** | **rotor** | **tube** | **centrifuga-tion force**  **(g)** | **°C** | **min** | **accel** | **deccel** |
| --- | --- | --- | --- | --- | --- | --- | --- | --- |
| cell | Eppendorf  5804 R | S-4-72 | Sarstedt 50 mL conical Ref. 62.559.001 | 300 | 20 | 10 | int. | int. |
| lEV |  |  |  | 2000 | 4 | 30 |  | slow |
| mEV 1. | Eppendorf  5810 R | F-34-6-38 | Falcon 50 mL conical  Ref. 352070 | 12,500 |  | 40 |  |  |
| mEV 2. | HERMLE Z 216 MK | Angle 220.87 | Eppendorf, 1.5 mL, PCR clean  Ref. 0030125.215 | 12,500 |  |  |  |  |

**Table S2. Parameters of NTA measurements**

|  | **mEV** |
| --- | --- |
| **sensitivity** | 60 |
| **shutter** | 100 |
| **frame rate** | 7.5 |
| **positions** | 11 |
| **cycles** | 2 |
| **min brightness** | 20 |
| **min size (nm)** | 5 |
| **max size (nm)** | 1000 |
| **temperature (°C)** | 25 |

**Table S3. Details of antibodies used in experiments**

| **antigen** | **reactivity** | **clonality** | **clone** | **isotype** | **dilution** | **fluoro-phore** | **manufactu-rer** | **cat. no.** |
| --- | --- | --- | --- | --- | --- | --- | --- | --- |
| CD81 | human | monoclonal | 5A6 | mouse IgG1 | 1:200 | PerCP-Cy5.5 | Sony Biotechnology | 2347540 |
| CD63 | human |  | H5C6 |  |  |  |  | 2365100 |
| - | - |  | MOPC21 |  |  |  |  | 2600750 |


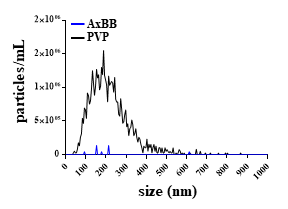


**Figure S1. Examination of blank PVP after dissolution with NTA**

(AxBB: Annexin Binding Buffer)


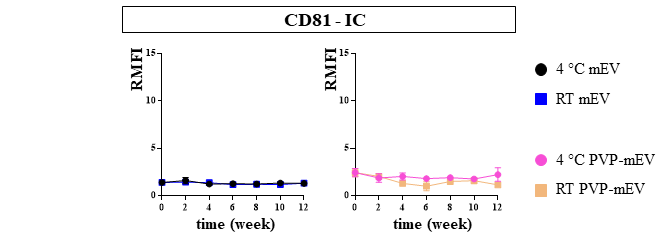


**Figure S2 Isotype control for CD81 staining**

(RMFI: relative median fluorescence intensity)


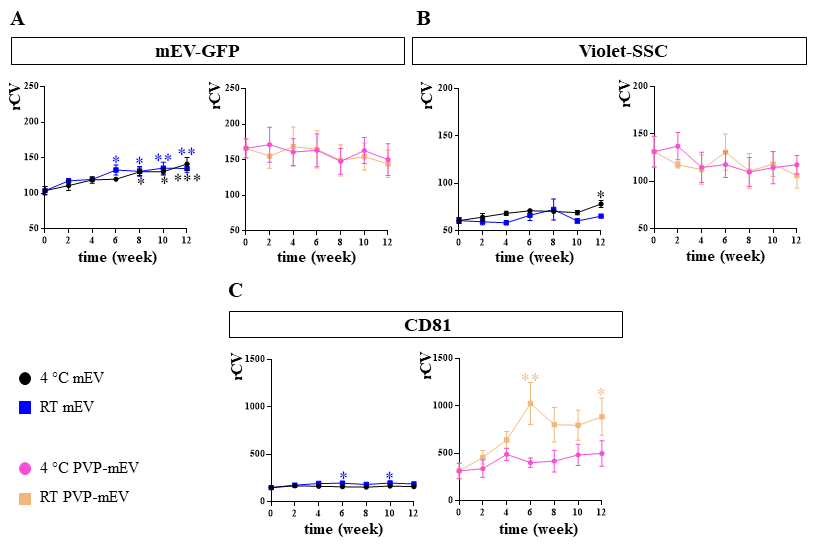


**Figure S3. Robust coefficient of variation (rCV) values ​​of GFP, Violet-SSC and CD81 parameters**
